# A Case Report of Brugada Syndrome Associated With Physical Trauma

**DOI:** 10.7759/cureus.55557

**Published:** 2024-03-05

**Authors:** Dinesh Nirmal, Nikola Stojanovic, Anandita Kishore, Shruthi Sivakumar, Asher Gorantla, Harshith Chandrakumar, Suzette Graham-Hill, Adam S Budzikowski

**Affiliations:** 1 Internal Medicine, State University of New York Downstate Health Sciences University, Brooklyn, USA; 2 Medicine, Brookdale University Hospital Medical Center, Brooklyn, USA; 3 Internal Medicine, Sisters of Charity Hospital, Buffalo, USA; 4 Neurology, State University of New York Downstate Health Sciences University, Brooklyn, USA; 5 Cardiology, State University of New York Downstate Health Sciences University, Brooklyn, USA; 6 Cardiology, Kings County Hospital Center, Brooklyn, USA; 7 Cardiovascular Medicine, State University of New York Downstate Health Sciences University, Brooklyn, USA

**Keywords:** brugada syndrome, brugada-like pattern, ventricular arrhythmia, cardiac sudden death, post trauma, brugada

## Abstract

Brugada syndrome is an autosomal dominant channelopathy that usually affects healthy young males without apparent structural heart disease. It is associated with a spectrum of variable and dynamic clinical manifestations, high risk of life-threatening ventricular arrhythmias, and sudden cardiac death. Our patient demonstrated transient and dynamic EKG changes of both type 1 (coved) and type 2 (saddleback) ST elevation, suggestive of the Brugada pattern that was associated with physical chest trauma and stressful situations. While common triggers like fever and certain drugs are well-recognized, this case illustrates the potential for physical stress and trauma to unmask or aggravate Brugada syndrome, albeit without definitive evidence for a causal link. Ultimately, this report underscores the importance of considering a broad differential diagnosis, including Brugada syndrome, in patients presenting with unexplained syncope or characteristic EKG changes, even when traditional triggers are absent.

## Introduction

Brugada syndrome is an autosomal dominant channelopathy with variable penetrance causing specific patterns on electrocardiogram (EKG), such as pseudo-right bundle branch block and ST-segment elevation (STE) in the right ventricular leads [1,2]. Asymptomatic patients with typical EKG findings have a Brugada pattern, whereas patients with characteristic EKG findings and other suggestive clinical manifestations have Brugada syndrome [3]. The significance of the Brugada syndrome lies in its prevalence in the population (between 0.1% and 1%), usually affecting healthy young males without apparent structural heart disease, variable and dynamic clinical manifestations, and high risk of life-threatening ventricular arrhythmias and sudden cardiac death [4,5]. Here, we describe a case of a healthy young male who developed Brugada syndrome after physical trauma.

## Case presentation

**Figure 1:**
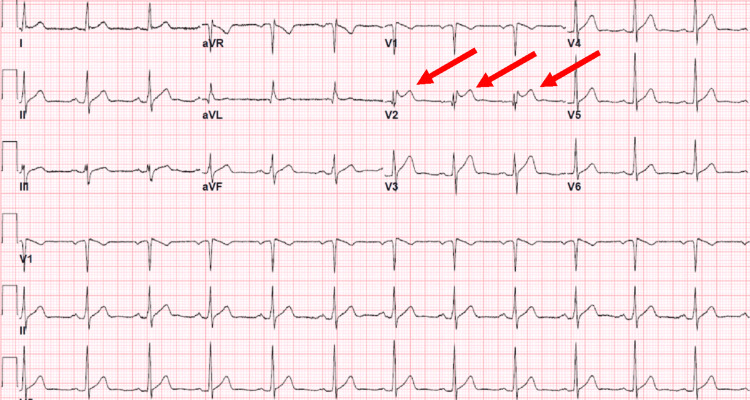
Electrocardiogram showing sinus rhythm and a typical saddleback pattern of ST elevation in the lead V2, suggestive of a type 2 Brugada pattern.

A 35-year-old male of Caribbean black ethnicity with no significant medical history presented to the emergency department (ED) because of chest pain after lifting a heavy object at work that suddenly popped out of his hands and caused a hit to the anterior chest wall. The chest pain was non-radiating and not associated with movement or exertion, and he did not experience palpitations or dyspnea. The pain was not relieved by nitroglycerin use. On further detailed review, the patient noted that he had a single unexplained episode of syncope at age 23, which was never worked up. His family history was negative for syncope, sudden cardiac death, nocturnal air gasping, or arrhythmias. The patient’s vitals remained within normal limits, and the physical exam was remarkable for mild tenderness to palpation of the left side of the chest. The standard 12-lead EKG showed sinus rhythm and a typical ST elevation saddleback pattern, most notably in the lead V2 and less notably in the lead V1, which suggests a type 2 Brugada pattern (Figure 1).

No prior EKG was available for comparison. The repeated standard 12-lead EKG showed the full resolution of the ST elevation, and the patient was treated conservatively with ibuprofen.

**Figure 2:**
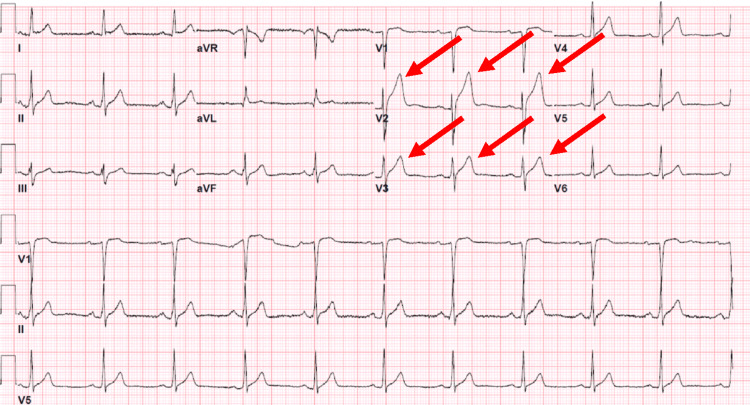
Electrocardiogram showing ST elevations in leads V2 and V3.

After nine months, the patient presented to the ED again with a similar complaint of mid-sternal chest pain after being involved in a minor motor vehicle accident in which he was the driver of a car being hit from the rear side, experiencing seatbelt-induced blunt trauma to the anterior chest. The chest pain was non-radiating, dull in quality, was not aggravated by exertion, and was not associated with shortness of breath, nausea, palpitations, or syncope. The physical exam noted tenderness over the sternal region on palpation without bruising, bleeding, or visible damage. The initial standard 12-lead EKG showed ST elevations in the leads V2 and V3 (Figure 2).

**Figure 3:**
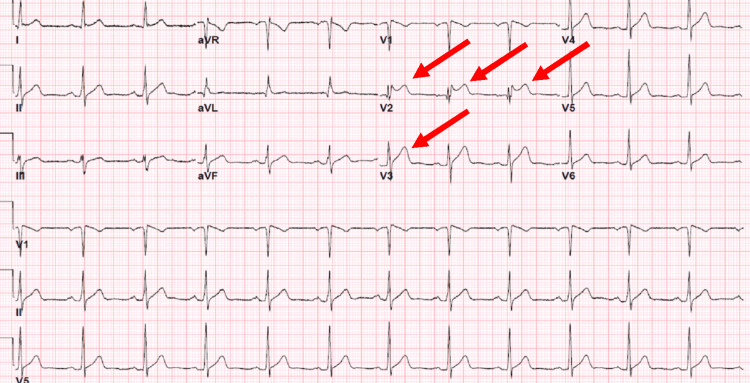
Electrocardiogram changes suggestive of a saddleback ST elevation in the lead V2 and partly V3 suggestive of a type 2 Brugada.

The complete blood count, comprehensive metabolic panel, serial measurements of troponin T, thyroid function panel, urinalysis, urine toxicology, and alcohol screen were negative. An echocardiogram showed a structurally intact heart with normal diastolic and systolic function and no valvular abnormalities. A computed tomography angiogram of the chest did not reveal an aneurysm or dissection. In the following days, the patient developed saddleback ST elevation in the leads V2 and V3 on the standard 12-lead EKG, suggestive of a type 2 Brugada pattern (Figure 3).

A treadmill stress test was negative. Stress echocardiography showed mild basal, inferoseptal, basal inferior, mid-inferoseptal, and apical septal hypokinesis, which prompted coronary angiography, which was negative for coronary artery disease. The inconsistency between the stress echocardiography results and the cardiac catheterization was considered to be a result of a false positive on the stress echocardiography. Fourteen-day Holter monitoring showed baseline sinus rhythm, with a heart rate ranging from 28 to 156 beats per minute, type I Mobitz atrioventricular block, and rare supraventricular ectopy and ventricular extrasystole (combined being <1% of heartbeats). No ventricular tachycardia, atrial fibrillation, or other arrhythmias were noted. The diagnosis of Brugada syndrome was made taking into account occasional Brugada EKG patterns and a history of an unexplained episode of syncope. The patient was informed of the diagnosis, and he declined a drug challenge to further specify his EKG findings after the discussion of the risks and benefits of the procedure. The patient and the medical team agreed to avoid triggers and ensure regular follow-up appointments with his primary care doctor and cardiologist. The patient's family members, being outside the US, were not available for a discussion on genetic testing. However, considering his hereditary condition, the patient was advised to inform his immediate relatives to consult a cardiologist in their home country.

## Discussion

Our patient demonstrated transient and dynamic EKG changes of saddleback ST elevation, suggestive of the type 2 Brugada pattern that was associated with physical trauma to the anterior chest wall and stressful situations [6]. The most common triggers for Brugada, such as fever, sodium channel blockers, tricyclic antidepressants, alcohol, cocaine, and electrolyte derangements, are well described [7,8]. Our patient did not have any of the well-documented triggers of Brugada syndrome.

The differential diagnoses were commotio cordis, pericarditis, and aortic dissection, but the patient had a relatively minor injury, mild chest wall tenderness, negative cardiac injury markers, and normal imaging findings. There is insufficient evidence to confirm a direct causative link between physical trauma and Brugada syndrome. However, stressful situations can disrupt the autonomic balance, crucial in developing Brugada syndrome. This is evident from the night-time occurrence of related tachyarrhythmias and the impact of autonomic tone alterations on typical ECG changes through pharmacological modulation.

A high index of suspicion is required to establish a diagnosis of Brugada syndrome, and careful risk stratification [9,10] is needed to properly determine further management, taking into account the elevated risk of syncope, arrhythmias, and sudden cardiac arrest and death. An individualized approach to the patient through shared decision-making and a close follow-up with an experienced cardiologist is required to minimize the risks.

## Conclusions

This case report highlights the complexities in diagnosing Brugada syndrome, an inherited channelopathy with a significant risk of syncope, ventricular arrhythmia, and sudden cardiac arrest in otherwise healthy individuals. Our patient exhibited dynamic and intermittent EKG changes suggestive of type 2 Brugada patterns in the context of mild anterior chest wall injuries. This case illustrates the potential for physical stress and trauma to unmask or aggravate Brugada syndrome in otherwise asymptomatic individuals. As in our patient, after the initial diagnosis is made, risk stratification with appropriate initial management can prevent sudden cardiac death at a young age from life-threatening arrhythmias.
